# Machine learning for prediction of newly diagnosed atrial fibrillation after emergency percutaneous coronary intervention during hospitalization in patients with acute ST-segment elevation myocardial infarction: a multi-center prospective study

**DOI:** 10.3389/fmed.2026.1864670

**Published:** 2026-07-29

**Authors:** Yazhu Wang, Yuehui Yin

**Affiliations:** 1 Department of Cardiology, The Second Affiliated Hospital of Chongqing Medical University, Chongqing, China; 2 Department of Cardiology, The Fifth People’s Hospital of Chongqing, Chongqing, China

**Keywords:** atrial fibrillation, gradient boosting, machine learning, percutaneous coronary intervention (PCI), risk prediction, ST-segment elevation myocardial infarction (STEMI)

## Abstract

**Background:**

In-hospital complications after emergency PCI in acute STEMI are important, especially newly diagnosed atrial fibrillation and are associated with hemodynamic instability, heart failure, stroke and mortality. There are complex nonlinear interactions between clinical, inflammatory, cardiac remodeling and procedural factors that traditional risk scores may not adequately capture. Thus, this study aimed to develop and validate an interpretable machine-learning model for early prediction of in-hospital NDAF in patients with acute STEMI undergoing emergency PCI.

**Methods:**

From January 2021 to December 2024, this study collected data on patients with acute STEMI after emergency PCI from the five tertiary general hospitals in Chongqing. Important clinical variables were identified using the selection operator and least absolute shrinkage. Based on the area under the curve, the best predictive model was selected from eight machine learning methods. The predictive model’s results were interpreted using Shapley Additive explanations.

**Results:**

Eighteen variables were chosen for model building, and a total of 154 patients with acute STEMI following emergent PCI were included. With the largest area under the curve of 0.991 (95% CI: 0.963–1), sensitivity of 0.846, specificity of 0.914, and Brier score of 0.2356, the Gradient Boosting model was selected. The Shapley value analytical framework was systematically applied to decode feature importance patterns and illuminate individual prognostic determinants

**Conclusion:**

The present study developed and compared eight machine learning models for the prediction of in-hospital NDAF among acute patients with STEMI treated with emergency PCI. The Gradient Boosting classifier achieved optimal predictive performance, highlighting its potential clinical utility for early risk stratification. By identifying high-risk individuals in advance, this model may support optimized clinical management and contribute to better prognosis in this high-risk population. Machine learning computations were implemented via a publicly accessible web server integrating the pre-trained Gradient Boosting prediction model. All analytical steps were performed following the platform’s default settings to maintain methodological standardization and reproducibility.

## Introduction

Acute ST-segment elevation myocardial infarction (STEMI) remains one of the leading causes of mortality and disability among cardiovascular patients worldwide (1). With the widespread use of emergency percutaneous coronary intervention (PCI) and the improvement of chest pain center systems, the acute-phase mortality of patients with STEMI has decreased significantly (2). However, successful revascularization is not entirely equivalent to improved clinical outcomes, and cardiovascular complications during hospitalization remain a key determinant of patient prognosis (3). Among these, NDAF (4) is one of the most common supraventricular arrhythmias in post-PCI patients with STEMI (5). NDAF during hospitalization has been reported in approximately 3.7–10.3% of patients with acute myocardial infarction or acute coronary syndrome, depending on the study population, diagnostic definition, and intensity of rhythm monitoring. This complication is clinically important because it is associated with heart failure, cardiogenic shock, stroke or systemic embolism, and increased mortality (6–8). Existing studies have also shown that NDAF after STEMI or acute coronary syndrome is associated with adverse in-hospital and long-term outcomes, including hemodynamic instability, heart failure, cardiogenic shock, stroke or systemic embolism, recurrent ischemic events, and increased all-cause mortality (3, 9, 10).

At present, the prediction of NDAF after STEMI in clinical practice mainly relies on traditional statistical models (11). Although previous studies have identified independent risk factors such as advanced age, Killip classification, heart rate, inflammatory markers, and left ventricular ejection fraction, and established classic evaluation tools including the CHADS_2_ or CHA_2_DS_2_-VASc scores, most of these methods are generalized regression models based on linear assumptions (12). In real clinical settings, the occurrence of NDAF after STEMI involves complex interactions among multiple pathophysiological mechanisms, such as ischemia-reperfusion injury, mitochondrial dysfunction, cardiomyocyte injury, autonomic dysfunction, inflammatory activation, and cardiac electrophysiological remodeling (13–15). Cardiometabolic myocardial injury, including diabetes-related cardiac remodeling, is also regulated by multiple interacting molecular pathways, supporting the need for multidimensional approaches in cardiovascular risk assessment (16).

Traditional regression models struggle to effectively capture the nonlinear relationships and implicit patterns embedded in these high-dimensional data, resulting in moderate predictive performance (C-statistic) that can hardly meet the requirements of individualized precision medicine (17). Machine learning and deep-learning approaches have provided a novel paradigm for clinical prediction, automated phenotyping, and precision management of cardiovascular and cardiometabolic disorders (18, 19). Compared with traditional models, machine learning algorithms have powerful feature-mining capabilities (20). They can automatically learn complex nonlinear interactions from massive clinical data, showing higher accuracy and stability in risk stratification and prognosis prediction of cardiovascular diseases (21–26). Nevertheless, machine learning-based prediction studies on in-hospital NDAF following emergency PCI in patients with STEMI are still relatively limited (27, 28). Most adopt a single-center retrospective design, which entails problems such as sample selection bias, insufficient external validation, and limited model generalizability. In addition, the performance comparison of different machine learning algorithms in this specific clinical scenario and the selection of the optimal model remains to be further explored.

Given these gaps, the present study was designed to provide a clearer clinical and methodological basis for early NDAF risk prediction in patients with acute STEMI undergoing emergency PCI. The justification for this work is threefold. First, NDAF during hospitalization is a clinically meaningful complication that may worsen short-term prognosis and therefore requires timely identification. Second, the development of NDAF after STEMI involves multiple interacting mechanisms, including ischemia-reperfusion injury, inflammation, neurohormonal activation, atrial remodeling, ventricular dysfunction, and procedural factors, which traditional linear prediction models may not adequately capture. Third, available prediction studies in this specific setting remain limited, and retrospective or single-center designs constrain many. Therefore, this multicenter prospective study integrated multidimensional clinical, laboratory, echocardiographic, and procedural data with interpretable machine learning algorithms to develop an early risk prediction model for in-hospital NDAF. By identifying high-risk patients before clinical deterioration, this model may support individualized rhythm monitoring, timely preventive strategies, and improved clinical decision-making after emergency PCI.

## 2 Materials and methods

### 2.1 Study design and setting

This study was designed as a multicenter prospective cohort investigation aimed at developing and validating a machine learning–based prediction model for NDAF in patients with acute ST-segment elevation myocardial infarction (STEMI) undergoing emergency percutaneous coronary intervention (PCI). The study was conducted in five tertiary care hospitals in Chongqing, China, and included patients enrolled over 4 years from January 2021 to December 2024. All patients received standardized clinical management, including emergency reperfusion therapy, antithrombotic treatment, secondary prevention, and in-hospital electrocardiographic monitoring, according to contemporary guideline-based protocols for the management of acute STEMI and myocardial revascularization (2, 3, 29). The primary objective was to integrate multidimensional clinical variables with advanced machine learning algorithms to create a precise, comprehensible model for early risk stratification of following (PCI) in STEMI.

### 2.2 Study population

#### 2.2.1 Inclusion criteria

(1) a known diagnosis of acute STEMI according to European Society of Cardiology (ESC) criteria, including typical ischemic symptoms, persistent ST-segment elevation on electrocardiography, and elevated cardiac biomarkers (30, 31); (2) has emergency percutaneous coronary intervention (PCI) within the index hospitalization; and To allow arrhythmic events to be accurately detected during hospitalization, (3) was monitored by continuous electrocardiographic (including telemetry, standard 12-lead ECG, or Holter monitoring) during hospitalization.

#### 2.2.2 Exclusion criteria

Patients were excluded if they met any of the following criteria:

(1) prior history of atrial fibrillation or other sustained arrhythmias;

(2) presence of significant structural heart disease, including severe valvular heart disease, congenital heart disease, or prior cardiac surgery;

(3) history of permanent pacemaker implantation;

(4) severe renal dysfunction, defined as estimated glomerular filtration rate (eGFR) < 30 mL/min/1.73 m^2^;

(5) advanced heart failure (Killip class IV) at presentation; or

(6) age < 18 years or > 80 years.

These criteria were applied to ensure a homogeneous study population and to minimize potential confounding factors that could influence the occurrence of atrial fibrillation or bias model development.

### 2.3 Outcome definition

The primary outcome of this study was the occurrence of in-hospital NDAF. NDAF was considered a case of 30 s or longer atrial fibrillation; this is rather reasonable based on the clinically and research-based criteria commonly used to diagnose AF. Continuous cardiac rhythm monitoring (telemetry, standard 12-lead electrocardiography (ECG) or Holter monitoring) was used to identify and prove the presence of episodes. The patients were constantly monitored with regard to electrocardiography, and this was monitored when they stayed in the hospital, so that they could be able to identify arrhythmic events. The presence or absence of NDAF was identified during discharge, and it was utilized as the major endpoint of the model development and validation.

### 2.4 Data collection and variables

Electronic medical records and hospital databases were used to construct a comprehensive multidimensional dataset for model development. This set of variables was a conglomeration of clinical, laboratory, imaging and procedural variables, which are manifestations of the multifactorial pathophysiology of atrial fibrillation in patients with STEMI.

### 2.4.1 Clinical variables

Clinical baseline was collected, including demographic variables (age and sex), and clinical comorbidities, such as hypertension, diabetes mellitus and smoking status. These variables were chosen because of previous evidence that showed the association of these variables with cardiovascular risk and arrhythmogenesis (6, 8, 32, 33).

#### 2.4.2 Laboratory parameters

A broad selection of laboratory biomarkers was involved to embrace cardiac damage, metabolic condition, and inflammatory reaction.

NT-proBNP, CK-MB, and cTnI are referred to as heart biomarkers, whereas NLRP3 inflammasome levels and CRP reveal the presence of inflammation. The lipid profile biomarkers include triglycerides, total cholesterol, LDL-C and HDL-C. Apelin-12 is a novel biomarker linked to atrial fibrillation and cardiovascular remodeling (34).

#### 2.4.3 Imaging variables

All patients underwent transthoracic echocardiographic evaluation, from which key structural and functional cardiac parameters were obtained. These included:

- Left atrial diameter
- Left ventricular end-diastolic diameter (LVD)
- Right ventricular diameter (RVD)
- Left ventricular ejection fraction (EF), assessed using the biplane Simpson’s method
- Cardiac output (CO)

These imaging parameters provided essential information regarding cardiac remodeling and hemodynamic status.

#### 2.4.4 Procedural variables

Procedural and perioperative features in the context of PCI were noted as well and included:

- Culprit coronary vessel: Involved in the infarction.
- Door-to-wire time (DtoW), the period between the onset of symptoms and successful guidewire passage through the occluded coronary artery.

These variables indicate procedural efficiency and ischemic burden, either of which can affect the risk of arrhythmia.

### 2.5 Data preprocessing

Before model development, standardized data preprocessing procedures were performed to improve data quality, enhance model robustness, and reduce potential bias. As clinical data is heterogeneous and multidimensional, a number of standardized preprocessing procedures were used. To begin with, the problem of missing data was solved by using the relevant imputation methods. Using k-nearest neighbors (KNN) imputation, continuous models with missing values were imputed, which conserves the underlying data distribution by using similarity between observations. This is because it has been extensively used in clinical machine learning research because of its capacity to minimize the loss of information and enhance the stability of the model. Second, data normalization and scaling were performed on all continuous variables to make them comparable across features and maximize the performance of machine learning algorithms that are sensitive to features. The standardization was done by converting variables to a mean and a standard deviation of one.

Third, outlier detection and management were carried out to reduce the effect of extreme values. Statistical techniques were employed to determine outliers (interquartile range (IQR) and clinical plausibility) and then managed by winsorization or exclusion as necessary. Finally, a hybrid resampling approach was used to deal with class imbalance among patients with and without NDAF. In particular, the Synthetic Minority Oversampling Technique (SMOTE) was used for data oversampling of the NDAF class in the training data and undersampling of the majority class to balance the outcome classes. The validation dataset was not altered; resampling was only performed on the training dataset after the 70:30 split to prevent information leakage and ensure that the class distribution of the validation data remained the same. This way, a model was learned from the minority class, and an unbiased evaluation of the model’s performance was achieved.

### 2.6 Feature selection

A systematic feature selection was carried out to cut dimensionalities and avoid overfitting of the model. To begin with, 41 candidate variables were added (including clinical, lab, imaging, and procedural data) due to a previous clinical understanding and the corresponding literature. The Least Absolute Shrinkage and Selection Operator (LASSO) regression, which is a regularization-based approach, was used to select the features, where coefficients of less informative variables were shrunk to zero to select the features. The optimal regularization parameter (λ) was estimated using k-fold cross-validation, which provides a good compromise between the complexity of the model and predictive accuracy. Based on the optimal λ (lambda. 1se criterion), 18 variables that had non-zero coefficients were selected as important predictors and later used in machine learning model development. This strategy was useful in minimizing redundancy, improving model interpretability, and decreasing the chances of overfitting.

### 2.7 Model development

In order to develop predictive models, the dataset was divided into a training set to develop the model and a validation set (30%) to test the performance of the model. Eight machine learning models, such as Logistic Regression, Decision Tree, Random Forest, Gradient Boosting, AdaBoost, Naive Bayes, XGBoost, and LightGBM, were used to improve the comparison of the model with the rest of the methods. Hyperparameter tuning was also carried out with 10-fold cross-validation in the training set to improve the performance and generalizability. The hyperparameters tested were: number of estimators, maximum tree depth, learning rate, minimum samples per split, minimum samples per leaf, regularization strength, and algorithm/kernel-specific parameters, which vary across model types. The hyperparameter set that yielded the best cross-validated performance was chosen and then tested in the validation cohort. The validation cohort was then tested with the optimized models. The current modeling approach was designed more as a prediction model to explore the rather small sample of NDAF events than as a model to be deployed. To prevent overfitting, LASSO regression was done before building the models, and optimization was done by 10-fold cross-validation. Of note, the validation conducted in this study was internal based on the training-validation split, and no independent external cohort existed. Thus, it is suggested that the reported model performance refers to internal validation performance only. The stability, generalizability and clinical applicability of the prediction model should be confirmed in larger and independent cohorts. Therefore, the reported model performance should be interpreted as internal validation performance only. Further validation in larger independent cohorts is required to confirm the stability, generalizability, and clinical applicability of the prediction model across different clinical settings and populations.

### 2.8 Model evaluation

The developed models were thoroughly evaluated in terms of different aspects, such as discrimination, calibration, and clinical utility, and adhered to the accepted reporting standards of predictive modeling. The receiver operating characteristic (ROC) curve analysis and area under the curve (AUC) were used to determine the discrimination capabilities of both models in discriminating between patients with and without NDAF.

The main classification measures were determined to determine model accuracy and clinical relevance, such as sensitivity, specificity, positive predictive value (PPV), and negative predictive value (NPV). This group of indicators provided a more accurate evaluation of the model functionality in diagnostics on different thresholds. Calibration curves and Brier score were used to measure the model calibration, i.e., the consistency of the predicted probabilities and measured results. Small Brier scores are also an indication that the probability of prediction is more predictive and reliable. The decision curves (DCA) were analyzed to determine the possible clinical relevance of the prediction models. Using this trade-off between true positives and false positives, DCA measures the net clinical value at a series of different threshold probabilities, therefore giving information about the usefulness of the model in practice in clinical decision-making.

### 2.9 Model interpretation

SHapley Additive exPlanations were used to evaluate the contribution of each feature to model predictions to enhance the transparency and interpretability of the model. A method based on game theory, SHAP offers both local and global interpretability. In particular, the mean absolute SHAP values were produced to rank feature importance, which allowed for determining the most impactful predictors. Moreover, SHAP summary plots were created to illustrate the direction and strength of feature effects throughout the dataset. Also, it was necessary to design single-level explanation plots (force plots) that would allow illustrating how single characteristics contributed to the outcomes of prediction in single patients. This interpretability model enhances the clinical believability and usefulness of the machine learning models.

### 2.10 Statistical analysis

Statistical calculations were conducted using R software (version 4.3.1) and Python (version 3.10). Continuous variables were first assessed for normality using the Shapiro-Wilk test. Normally distributed variables were presented as mean ± standard deviation and compared using Student’s *t*-test, whereas non-normally distributed variables were presented as median with interquartile range and compared using the Mann-Whitney U test. Categorical variables were analyzed with the chi-square (χ^2^) test, presented as counts and percentages. A *P*-value of less than 0.05 indicated statistical significance in all two-sided tests.

### 2.11 Ethical considerations

Ethical approval for the study protocol was obtained from the Ethics Committee of Chongqing Fifth People’s Hospital (Approval No.: 2021CQSDWRMYYEC-020). All patients provided written informed consent prior to inclusion in the study. Patient confidentiality and data privacy were strictly maintained throughout the research process.

## 3 Results

### 3.1 Baseline characteristics of the study population

A total of 154 patients with acute ST-segment elevation myocardial infarction (STEMI) who underwent emergency percutaneous coronary intervention (PCI) were included in the final analysis. Among them, 115 patients (74.68%) maintained sinus rhythm (SR), whereas 39 patients (25.32%) developed NDAF during hospitalization. All NDAF events were identified during the index hospitalization based on the predefined rhythm-monitoring strategy described in the Methods section. Table 1 summarizes the study population’s baseline clinical, laboratory, echocardiographic, and demographic parameters. NDAF patients were substantially older (66.92 ± 10.37 vs. 60.45 ± 12.05 years, *P* = 0.0018) and had a smaller percentage of female patients (*P* = 0.040) than patients in the SR group. The NDAF group had improved levels of total cholesterol (*P* = 0.0007) and low-density lipoprotein cholesterol (LDL-C) (*P* = 0.0181), as well as considerably lower blood albumin (*P* = 0.0031).

**TABLE 1:** Baseline clinical, laboratory, and echocardiographic characteristics of patients with sinus rhythm (SR) and NDAF.

Table parameters	Baseline (*n* = 154)	SR (*n* = 115)	NDAF (*n* = 39)	*P*-value
Age, y	62.09 ± 11.95	60.45 ± 12.05	66.92 ± 10.37	0.0018
Female sex, *n*(%)	35/154 (22.7)	21 /115(81.3)	14/39 (35.9)	0.040
Smoking, *n*(%)	108/154 (70.1%)	84/115 (73.0%)	24/39 (61.5%)	0.2484
Hypertension, n (%)	93/154 (60.4%)	72/115 (62.6%)	21/39 (53.8%)	0.4369
Diabetes, *n*(%)	52/154 (33.8%)	43/115 (37.4%)	9/39 (23.1%)	0.1506
Culprit vessel, *n*(%)	88/154 (57.1%)	64/115 (55.7%)	24/39 (61.5%)	0.6493
Culprit vessel (LCX), *n*(%)	12/154 (7.8%)	8/115 (7.0%)	4/39 (10.3%)	0.5007
Culprit vessel, *n*(%)	54/154 (35.1%)	43/115 (37.4%)	11/39 (28.2%)	0.3983
Uric acid, μmol/L	362.69 ± 110.37	368.32 ± 95.97	346.07 ± 145.05	0.3757
Random glucose, mmol/L	9.20 ± 4.15	9.11 ± 4.25	9.45 ± 3.87	0.6492
Albumin, g/L	38.99 ± 4.83	39.60 ± 4.96	37.20 ± 3.96	0.0031
Globulin, g/L	28.13 ± 4.81	28.41 ± 4.82	27.31 ± 4.76	0.2216
Hemoglobin, g/L	139.83 ± 18.96	140.78 ± 20.32	137.03 ± 14.06	0.2047
HDL-C, mmol/L	1.09 ± 0.31	1.10 ± 0.31	1.09 ± 0.29	0.8610
LDL-C, mmol/L	3.02 ± 0.83	2.81 ± 0.51	3.09 ± 0.91	0.0181
Triglyceride, mmol/L	1.30 ± 1.13	1.32 ± 1.26	1.22 ± 0.61	0.5239
TC, mmol/L	4.79 ± 2.14	4.06 ± 1.12	5.04 ± 2.34	0.0007
Creatinine, μmol/L	82.85 ± 40.28	78.23 ± 26.20	96.47 ± 64.95	0.0951
Urea, nitrogen, mmol/L	6.65 ± 7.09	5.86 ± 1.88	8.99 ± 13.58	0.1597
LAD, mm	32.38 ± 5.37	30.77 ± 3.78	37.13 ± 6.50	< 0.001
LVD, mm	45.34 ± 6.39	44.56 ± 5.45	47.67 ± 8.23	0.0324
RAD, mm	31.17 ± 4.73	30.57 ± 4.41	32.95 ± 5.25	0.0136
RVD, mm	20.56 ± 4.55	19.12 ± 2.00	24.82 ± 6.83	< 0.001
IVS, mm	10.44 ± 1.86	10.31 ± 1.87	10.83 ± 1.79	0.1249
LVPW, mm	8.93 ± 1.42	8.87 ± 1.38	9.11 ± 1.54	0.3796
AAO, mm	28.96 ± 2.84	29.24 ± 2.65	28.13 ± 3.22	0.0562
MPA, mm	18.86 ± 1.93	18.86 ± 1.97	18.85 ± 1.86	0.9665
SV, mL	49.91 ± 13.67	48.76 ± 13.27	53.31 ± 14.43	0.0876
EF, %	51.03 ± 10.77	52.49 ± 10.96	46.72 ± 8.99	0.0016
CO, mL/min	3322.75 ± 1568.08	3716.59 ± 1076.91	1535.33 ± 2172.37	< 0.001
DtoW, min	408.90 ± 597.19	359.35 ± 527.14	555.00 ± 756.94	0.1408
cTnI, ng/mL	0.80 (0.10–9.89)	0.80 (0.10–7.78)	0.62 (0.13–20.79)	0.4178
CKMB, ng/mL	22.70 (3.40–100.00)	29.84 (3.85–100.00)	6.64 (2.51–69.38)	0.1881
MYO, ng/mL	196.09 (32.26–390.48)	195.17 (30.84–398.83)	212.71 (50.52–362.42)	0.9602
CRP, mg/L	4.08 (1.34–12.85)	3.38 (1.15–10.40)	9.50 (2.42–27.24)	0.0092
D-Dimer, μg/mL	0.24 (0.11–0.64)	0.20 (0.10–0.46)	0.62 (0.27–0.98)	< 0.001
Apelin_12, ng/mL	0.49 (0.20–0.57)	0.54 (0.43–0.64)	0.16 (0.13–0.21)	< 0.001
NLRP3, ng/mL	0.32 (0.21–0.43)	0.23 (0.14–0.34)	0.34 (0.21–0.48)	0.0005
NTproBNP, pg/mL	688.66 (294.43–2131.60)	510.70 (241.70–1016.65)	2706.00 (1175.30–4767.32)	< 0.001

Values are presented as mean ± standard deviation for normally distributed continuous variables, median (interquartile range) for non-normally distributed continuous variables, or n (%) for categorical variables. Normality was assessed using the Shapiro-Wilk test. Between-group comparisons were performed using Student’s *t*-test, Mann-Whitney U test, or chi-square test, as appropriate. SR, sinus rhythm; NDAF, newly diagnosed atrial fibrillation.

Patients in the NDAF group demonstrated significantly greater left atrial diameter, left ventricular diameter (LVD), right atrial diameter (RAD), and right ventricular diameter (RVD) due to structural cardiac remodeling (*P* < 0.05). They also experienced worsened cardiac function, indicated by reduced cardiac output (CO) (*P* < 0.001) and left ventricular ejection fraction (EF) (*P* = 0.0016). Furthermore, markers of thrombosis, inflammation, and heart stress were notably elevated, including NT-proBNP, C-reactive protein (CRP), NLRP3 inflammasome, and D-dimer (all *P* < 0.01). Conversely, Apelin-12 levels were significantly lower in this group (*P* < 0.001). No significant differences were found between the NDAF and control groups regarding culprit vessels, diabetes mellitus, hypertension, smoking status, or common laboratory indicators (all *P* > 0.05).

### 3.2 Feature selection using LASSO regression

To identify the most relevant predictors associated with NDAF. A total of 41 candidate variables, encompassing clinical, laboratory, imaging, and procedural parameters, were initially included in the analysis.

**FIGURE 1:**
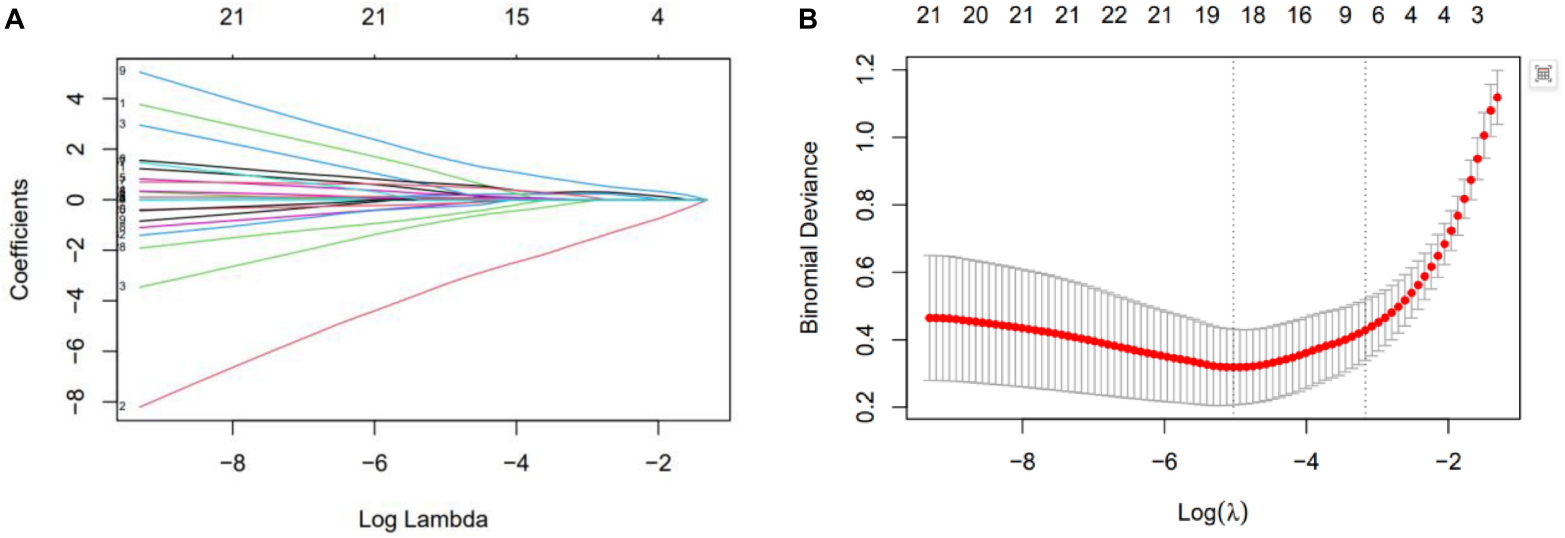
Feature selection using LASSO regression. **(A)** Coefficient profiles of candidate variables as a function of the regularization parameter (λ). **(B)** Ten-fold cross-validation for optimal λ selection, with the vertical dashed line indicating the λ.1se value used for model construction.

The penalization effect of the LASSO method was observed in the profile of the coefficients of all candidate variables, which progressively went towards zero with an increase in the regularization parameter (λ), as shown in Figure 1. Ten-fold cross-validation was done to identify the best regularization, and the resultant cross-validation curve. The best model was chosen based on the lambda.1se criterion (λ = 0.00653), that is, balancing the simplicity of the model against the predictive performance.

This method reduced 18 variables that had non-zero coefficients to the final ones used as the main predictors to be used in the development of the model. These variables included Apelin-12, left atrial diameter, right ventricular diameter (RVD), N-terminal pro–B-type natriuretic peptide (NT-proBNP), NLRP3, left ventricular diameter (LVD), creatine kinase-MB (CK-MB), door-to-wire time (DtoW), stroke volume, age, total cholesterol, triglycerides (TG), high-density lipoprotein cholesterol (HDL-C), creatinine, urea nitrogen, ascending aorta diameter (AAO), globulin, and female sex. It is important to note that some of these features exhibited significant contributions to the model, especially Apelin-12, LAD, RVD, NT-proBNP, and NLRP3, which may indicate the importance of these features in the pathophysiological mechanisms of NDAF. The LASSO-based feature selection was efficient in terms of dimensionality reduction of data, minimization of multicollinearity and further enrichment of the interpretability and robustness of the resulting machine learning models.

### 3.3 Development and comparison of machine learning models

To evaluate the predictive performance of different machine learning approaches, a total of eight models were developed and compared, including Logistic Regression, Decision Tree (DT), Random Forest (RF), Gradient Boosting, Naïve Bayes (NB), AdaBoost, XGBoost, and Light Gradient Boosting Machine (LightGBM).

**FIGURE 2:**
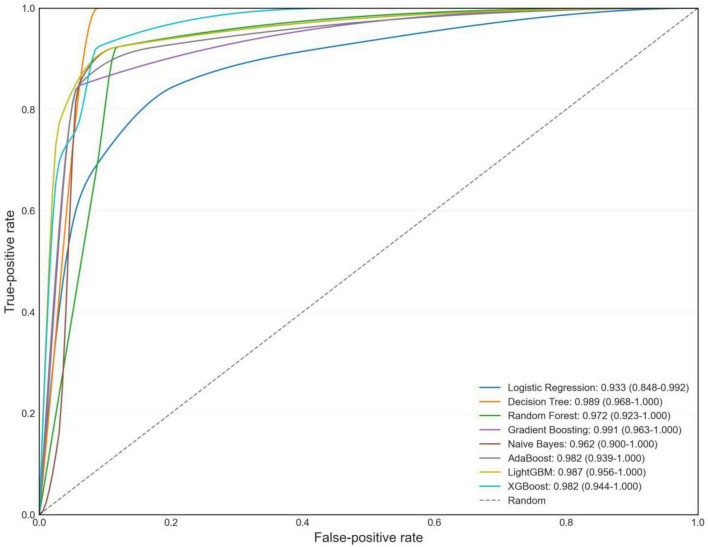
ROC curves of eight machine learning models for predicting NDAF.

These models were evaluated based on the (ROC) curve, as shown in Figure 2. The Brier score of the Gradient Boosting model was 0.2356, indicating good overall calibration and reliable probabilistic prediction.

The other machine learning models also demonstrated good predictive performance, albeit a little bit less than the Gradient Boosting model. In particular, the AUC values included 0.933 (Logistic Regression), 0.989 (Decision Tree), 0.972 (Random Forest), 0.962 (Naive Bayes), 0.982 (AdaBoost), 0.987 (LightGBM), and 0.982 (XGBoost). Although the ensemble-based models performed well compared with the conventional statistical models, none of them performed better than the Gradient Boosting algorithm. These results indicate that although several machine learning models have the potential to predict NDAF in patients with acute STEMI following PCI, the Gradient Boosting model shows better performance in the discriminatory criterion and, therefore, is the best model to continue analyzing and using in the clinical environment.

### 3.4 Model performance evaluation

The performance of the machine learning models was evaluated based on discrimination, calibration, and clinical utility. All models showed strong predictive performance, with AUC values over 0.93. Based on the validation cohort, the Gradient Boosting model achieved a sensitivity of 0.846, specificity of 0.914, positive predictive value of 0.786, and negative predictive value of 0.941. The corresponding 95% confidence intervals are shown in Table 2. Other models, including Random Forest (AUC = 0.972), AdaBoost (AUC = 0.982), XGBoost (AUC = 0.982), and LightGBM (AUC = 0.987), performed well but were outperformed by Gradient Boosting.

**TABLE 2:** Diagnostic performance of the gradient boosting model in the validation cohort.

Performance metric	Estimate	95% confidence interval
AUC	0.991	0.963–1.000
Sensitivity	0.846	0.578–0.957
Specificity	0.914	0.776–0.973
Positive predictive value	0.786	0.524–0.924
Negative predictive value	0.941	0.812–0.984
Brier score	0.2356	Not applicable

**FIGURE 3:**
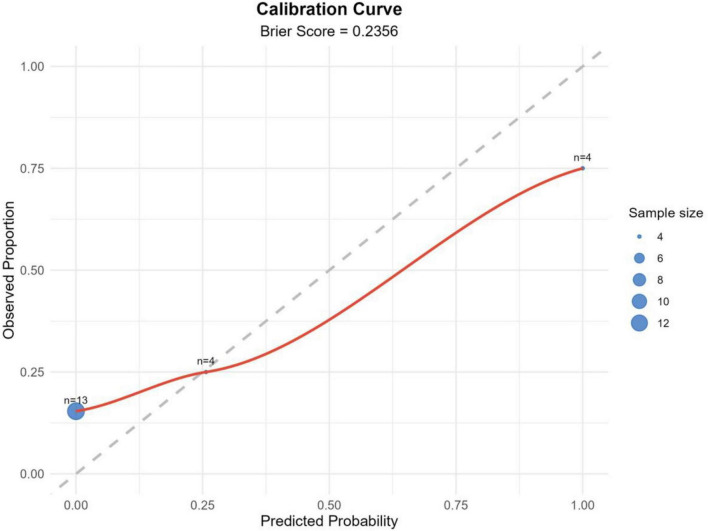
Calibration curve of the gradient boosting model for predicting NDAF.

Calibration performance of the Gradient Boosting model was further assessed using a calibration curve. The results were well balanced to the predicted probabilities, with the curve of calibration being approximate to the desired diagonal reference line, as observed in Figure 3. The result suggests that there was a good correspondence between the predicted and actual risk of NDAF. In addition, the low Brier score of 0.2356 further supported the reliability and accuracy of the Gradient Boosting model in estimating the probability of NDAF.

**FIGURE 4:**
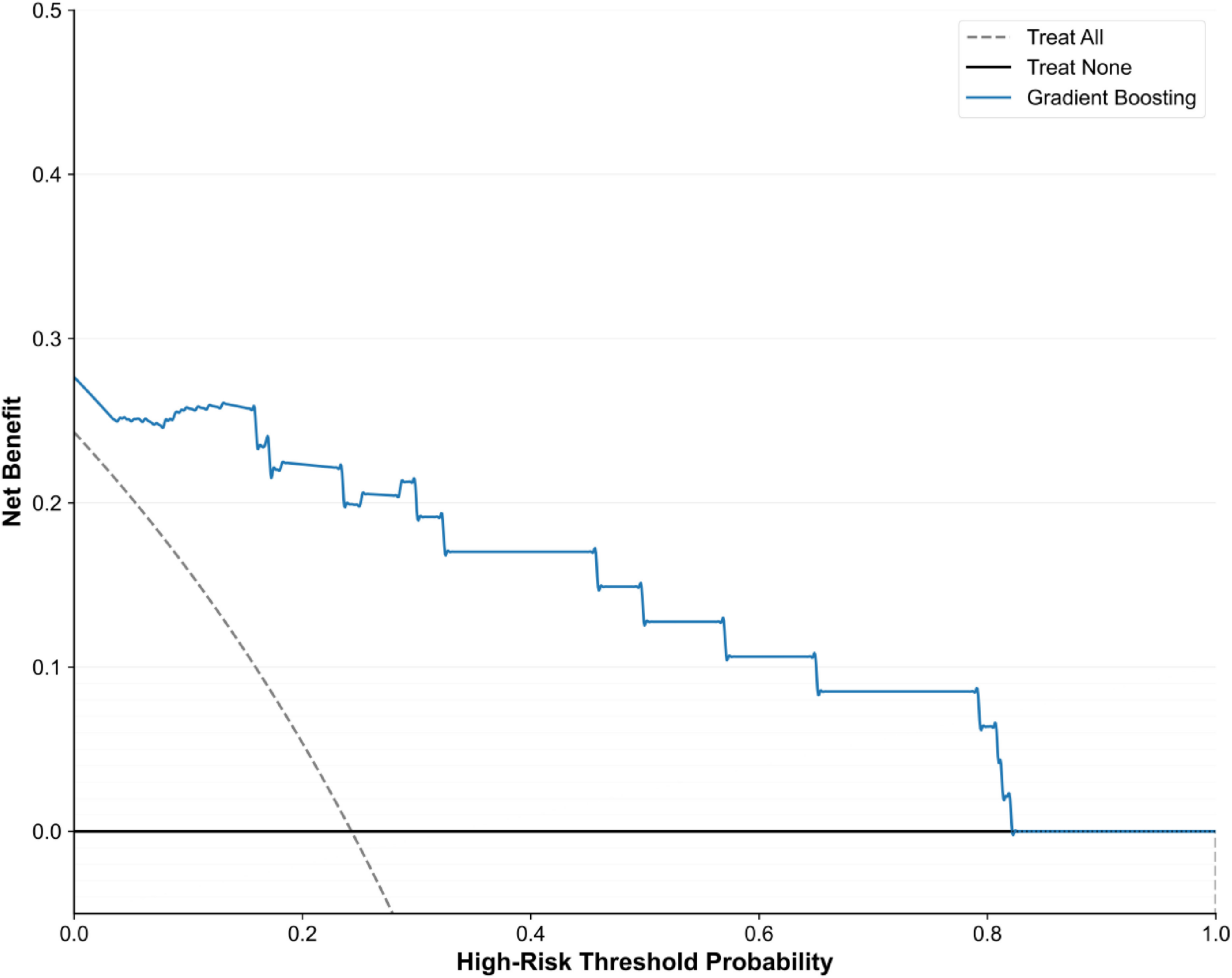
Decision curve analysis of the gradient boosting model for predicting NDAF.

The gradient Boosting model had a greater net clinical benefit at a large range of threshold probabilities than either the treat-all or treat-none approaches, as shown in Figure 4. This means that the model has considerable clinical implications, and it may assist in recognizing Patients at risk who could be assisted by early intervention or increased monitoring. Collectively, these results suggest that not only did the Gradient Boosting model attain a high discrimination and calibration level, but it can be used clinically to make the most predictions in relation to NDAF in post-STEMI patients post-PCI.

### 3.5 Model Interpretation using SHAP analysis

**FIGURE 5:**
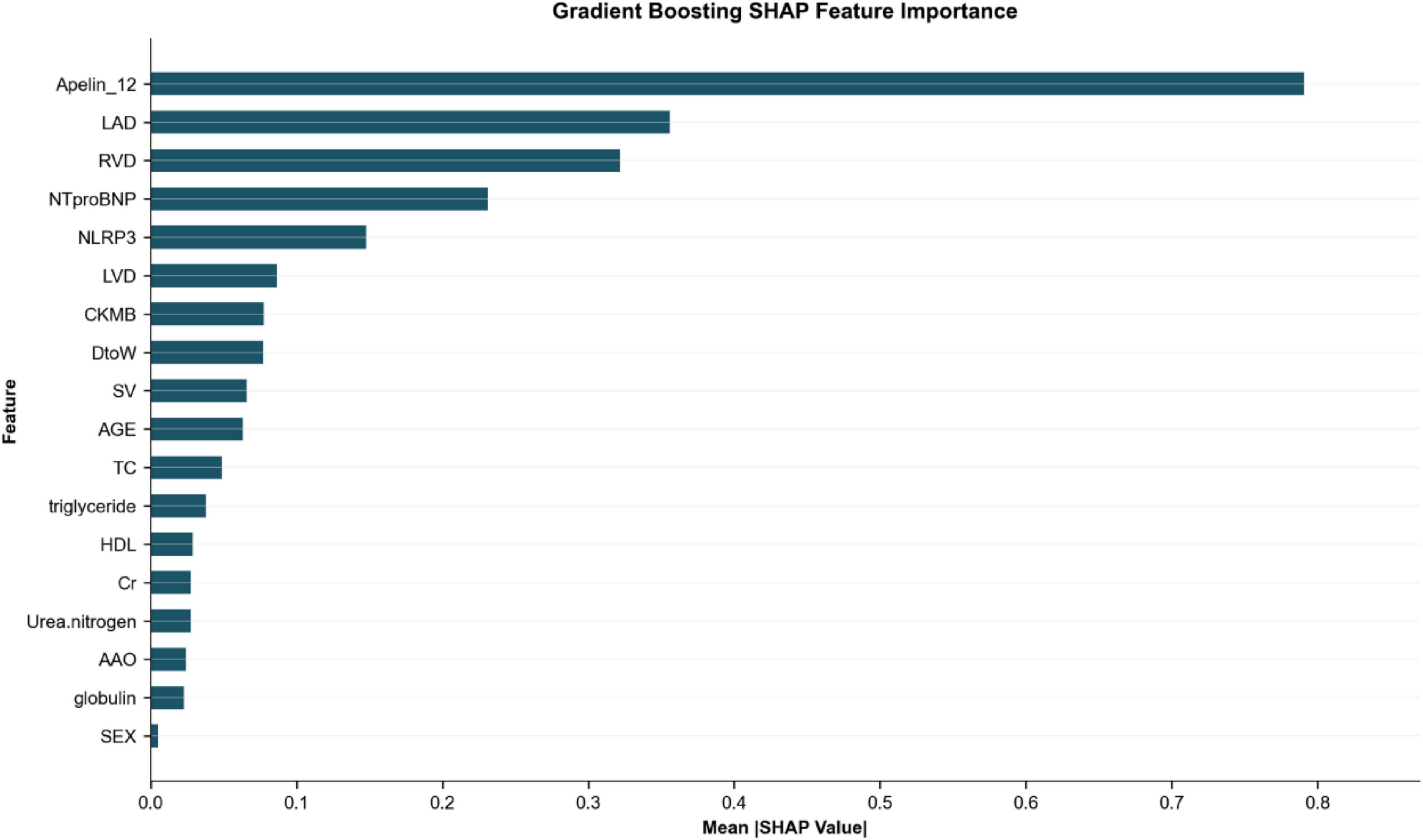
SHAP feature importance of the gradient boosting model for predicting NDAF.

SHapley Additive exPlanations analysis was used to measure each feature’s contribution to the prediction in order to improve the machine learning model’s interpretability. Figure 5 shows that Apelin-12 was the most significant predictor using the mean absolute values of SHAP, then left atrial diameter, right ventricular diameter (RVD), N-terminal pro-B-type natriuretic peptide (NT-proBNP), and NLRP3. The results indicate the significant contributions of inflammatory processes, activation of neurohormones, and structural remodeling of the heart in the pathophysiology of NDAF.

**FIGURE 6:**
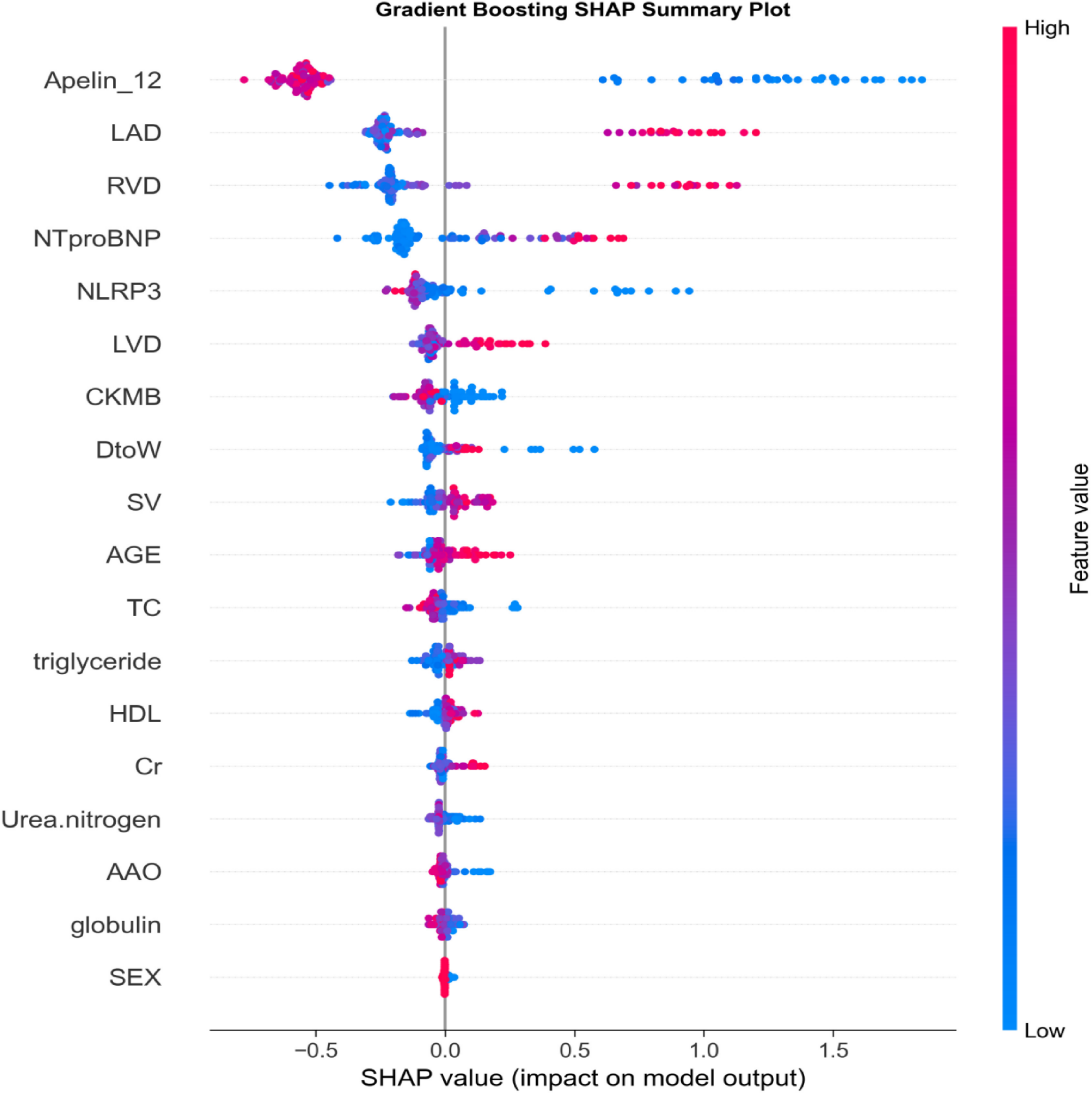
SHAP summary plot showing the distribution and direction of feature effects on NDAF prediction.

The overall influence of every feature in the model output is also revealed in the SHAP summary plot (Figure 6). An increased risk of NDAF was related to higher values of LAD, RVD, NT-proBNP and NLRP3 but not to higher values of Apelin-12, indicating its possible protective effect. This tendency coincides with the known pathophysiology of atrial fibrillation.

**FIGURE 7:**
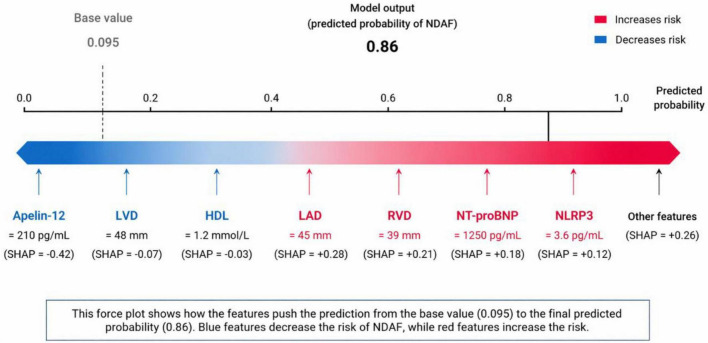
SHAP force plot illustrating the contribution of individual features to the predicted risk of NDAF in a representative patient.

In addition, the prediction at an individual level was also explained using SHAP force plots as shown in Figure 7. These plots are also used to demonstrate the overall contribution of the individual features to the final outcome of the prediction on a single patient and demonstrate the additive effect of risk-enhancing and risk-reducing variables. This interpretability boosts the transparency of the model and may aid its possible use in personalized clinical decisions. SHAP analysis proved that the Gradient Boosting model is not only very accurate but also clinically interpretable, allowing one to identify essential predictors and gain a better insight into the mechanisms that may be related to NDAF in patients with STEMI undergoing PCI.

## 4 Discussion

In the current multicenter prospective study, 39 patients (25.32%) of 154 patients with acute STEMI who underwent emergency PCI had developed NDAF during the hospital stay. This rate is higher than the 3.7–17.1% observed in many AMI or ACS cohorts, perhaps due to the high-risk STEMI population, the stress of an acute ischemia and hemodynamic stress and the prospective in-hospital rhythm monitoring. The use of telemetry, standard 12-lead ECG or Holter monitoring may have led to a greater detection of transient and asymptomatic AF episodes. Variations in patient selection, the extent of monitoring, diagnostic criteria and the timing of rhythm assessment can also play a role in the differences between the studies. Thus, while the observed incidence is suggestive of clinical relevance of NDAF in this situation, the incidence should be viewed with caution and should be confirmed in larger independent cohorts.

In the present study, eight machine learning models were developed to predict in-hospital NDAF among patients with acute STEMI undergoing emergency PCI. The Gradient Boosting model showed the best discrimination, with an AUC of 0.991, and outperformed Logistic Regression, Decision Tree, Random Forest, Naïve Bayes, AdaBoost, XGBoost, and LightGBM. This finding suggests that ensemble-based nonlinear algorithms may be better suited than conventional linear approaches for capturing the complex relationships among clinical, inflammatory, echocardiographic, and procedural factors involved in NDAF development. Previous studies have also shown that risk prediction models incorporating multiple clinical and biomarker variables can improve identification of patients at risk for new-onset AF after AMI or PCI, although reported model performance has varied across populations and modeling strategies (32, 35, 36).

Compared with previous prediction studies, our model demonstrated higher apparent discrimination. Yang et al. developed a prediction model for new-onset AF in patients with acute myocardial infarction after emergency PCI. They reported that inflammatory indices, age, left atrial diameter, Killip class, and NT-proBNP were important predictors (35). Wu et al. also reported that age, left atrial diameter, systemic inflammatory response index, creatinine, and the triglyceride-glucose index were associated with new-onset AF after AMI and PCI, with a prediction model AUC of 0.780 (36). Similarly, Bi et al. identified age, hypertension, troponin I, and left atrial diameter as independent predictors of new-onset AF during hospitalization in AMI patients (32). Our findings are consistent with these studies in showing that atrial structural remodeling, cardiac stress, inflammation, and baseline clinical characteristics are central to the development of NDAF. However, our study differs by integrating echocardiographic remodeling variables, inflammatory biomarkers, Apelin-12, and machine learning-based SHAP interpretation in a multicenter prospective STEMI cohort.

However, the high predictive performance of the Gradient Boosting model should be cautioned as the sample size was relatively small and the number of NDAF events was limited. This study was a multicenter prospective design, but only 154 patients and 39 NDAF events were available for developing and validating this model. Thus, the very high AUC might in part reflect optimism or overfitting due to internal validation. To reduce this risk, we used LASSO-based feature selection to reduce dimensionality of the predictors, cross-validation during model optimization, and evaluation of model performance in a separate validation subset. However, this current study did not have a separate external validation cohort. Hence, the results of the Gradient Boosting model should be seen as internal validation results only. The validity of the model for other hospitals, patient groups, monitoring schemes, and laboratories is unknown. Independent large external cohorts should be used to validate the model before it can be routinely used for clinical decision making. However, the current model is useful to explore a framework for early identification of high-risk patients who may benefit from intensified monitoring of arrhythmias, more detailed assessment of hemodynamics and prompt action following emergency PCI.

The use of SHAP analysis further improved the clinical interpretability of the model. Rather than only reporting classification performance, SHAP allowed us to identify the direction and relative contribution of predictors. The most influential variables in our model—Apelin-12, left atrial diameter, right ventricular diameter, NT-proBNP, and NLRP3—are biologically plausible and align with prior evidence linking atrial remodeling, ventricular dysfunction, neurohormonal activation, and inflammation with AF development (37–43). Therefore, the model does not merely provide a statistical prediction but also reflects clinically meaningful pathophysiological mechanisms.

SHAP analysis revealed that Apelin-12, left atrial diameter, right ventricular diameter, NT-proBNP, and NLRP3 were the five most influential predictors in the Gradient Boosting model. These variables are clinically plausible, as they represent complementary biological domains in the development of AF, such as atrial structural remodeling, ventricular dysfunction, neurohormonal activation, inflammation and myocardial fibrosis (44). This confirms that NDAF after STEMI is a complex phenomenon and not only dependent on one factor but on the interaction of factors such as ischemic injury, hemodynamic stress, atrial substrate vulnerability, and systemic inflammation.

In our model, the most important feature was Apelin-12, and lower levels of Apelin-12 were associated with higher predicted NDAF risk. This is consistent with the previous clinical evidence of decreased levels of apelin in patients with atrial fibrillation (37). Experimental data have also indicated that apelin can prevent atrial fibrosis and susceptibility to AF by inhibiting the TGF-β/Smad2/α-SMA pathway (38). Furthermore, clinical studies have identified decreased Apelin levels to be associated with AF recurrence after rhythm-control interventions (39). Thus, the role of Apelin-12 in our model could be indicative of the involvement of Apelin-12 in atrial fibrosis, myocardial remodeling and arrhythmogenic susceptibility following an acute ischemic stimulus (45). Nevertheless, Apelin-12 is less studied in the field of post-STEMI NDAF, and this finding should be considered hypothesis-generating and needs to be validated in larger prospective cohorts.

Left atrial diameter was yet another significant predictor, similar to previous studies of AMI and AF that have found enlargement of the LA to be an important marker of atrial remodeling, and by proxy, AF risk (32, 35, 36, 46–49). Enlargement of the left atrium may be a consequence of chronic increased filling pressure, atrial stretch, fibrosis, and electrical remodeling, all of which can facilitate initiation and maintenance of AF. Left atrial size is a clinically relevant predictor of NDAF, and this size may be exacerbated by ischemia, increased left ventricular filling pressure and neurohormonal activation in the acute STEMI setting.

The diameter of the right ventricle also played a large part in the model. This finding could suggest that left-sided atrial remodeling is not the only factor to determine NDAF risk following STEMI, but the global cardiac structural and hemodynamic disturbance as well. Prior MESA-RV data have demonstrated an association between right ventricular structure and function and the incidence of AF (40). RVE may be a marker of ischemic load, pulmonary pressure changes, reduced ventricular interaction or greater hemodynamic stress, and might be associated with vulnerability to atrial arrhythmias in patients with STEMI.

Similarly, NT-proBNP proved to be an important determinant in our model and is known to be associated with an increased risk of AF (41). NT-proBNP is a marker of myocardial wall stress, myocardial dysfunction and intracardiac pressure. In STEMI, elevated NT-proBNP can reflect greater infarct size, worse ventricular function, and/or higher atrial pressures, all of which have the potential to lead to atrial stretch and electrical instability. SHAP also identifies NT-proBNP, and this finding aligns with prior studies looking at predicting AMI that included NT-proBNP and found a relation to new onset AF post PCI (35).

Finally, NLRP3 also played a significant role in the prediction of NDAF, further supporting the role of inflammation in the pathogenesis of AF. It has been demonstrated that activation of the NLRP3 inflammasome leads to atrial electrical remodeling and remodeling of atrial structure, and is important in the pathogenesis of AF (42, 43). Ischemia-reperfusion injury and systemic inflammatory activation can promote inflammasome signaling, leading to increased atrial vulnerability during acute STEMI. Thus, the addition of NLRP3 to the model also reinforces the biological plausibility of a role for inflammatory activation as a connecting pathway between acute myocardial injury and subsequent atrial arrhythmogenesis.

In general, these results indicate that the Gradient Boosting model is capable of combining various pathophysiological aspects instead of just one predictor. Concurrently, Apelin-12, left atrial diameter, right ventricular diameter, NT-proBNP and NLRP3 are all associated with NDAF after emergency PCI, suggesting that structural remodeling, hemodynamic stress, neurohormonal activation and inflammation are all important factors. This simultaneous interpretation offers more scientific underpinning for the model. It could be useful for clinicians to comprehend why some patients with STEMI are more susceptible to NDAF in the hospital.

The proposed model is clinically aimed to be used to stratify early risk in the index hospital after emergency PCI. From the practical standpoint, prediction might be made following PCI if all baseline clinical data, admission laboratory values, echocardiographic measurements, and procedural data are known. A great majority of the variables used in the model could be derived from the routine in-hospital evaluation, including age, sex, lipid profile, renal function markers, echocardiographic parameters and door to wire time, NT-proBNP. But Apelin-12 and NLRP3 are not often ordered in many labs, which could reduce the use of the entire model in clinical practice right now. Accordingly, future studies should examine the feasibility of biomarker measurement, external validation of the model, and the possibility of creating simpler models, using routinely measured predictors, prior to routine clinical use. Beyond this, novel cardiometabolic treatments such as GLP-1 receptor agonist based therapies could have implications for heart failure biology and cardiovascular risk modification, but further study is needed to better understand the connection between emerging cardiometabolic treatments and NDAF after STEMI (50).

Moreover, we have plotted the DCA curves to verify the excellent clinical utility of the Gradient Boosting model. All of these results prove that the model for newly diagnosed AF is a valid tool to identify high-risk newly diagnosed AF patients but also provides a tangible basis to inform clinical decision-making, which has the potential to streamline treatment planning and, eventually, improve patient outcomes. Although this study has achieved significant results, there are still some limitations. To start with, the sample size of clinical cases in this research is very small, and thus, there is a likelihood of statistical bias in the analysis of data. Future studies need to further expand the sample size to evaluate the accuracy and discriminative power of the model. Secondly, the analysis did not cover some of the possible factors that could be related to the incidence of NDAF in hospitalized patients with STEMI, potentially impacting the fidelity of the prediction model. Thirdly, this study did not include follow-up data and only analyzed short-term data during the patient’s hospitalization. Long-term follow-up data will be introduced in the future to give further analysis of its clinical significance. Future prediction models may also consider lifestyle-related and chronobiological factors, because circadian rhythm and metabolic regulation have been increasingly linked with cardiovascular health and disease prevention (51).

## 5 Conclusion

In this multicenter prospective study, we developed and validated a machine learning–based model for predicting in patients with acute STEMI undergoing emergency PCI. The Gradient Boosting model with eight algorithms presented a better predictive model with great discrimination, calibration, and clinical utility compared to the other eight algorithms. The incorporation of multidimensional clinical data greatly improved the accuracy of models when compared to conventional methods. SHAP analysis additionally enhanced understanding of the models by determining the most significant predictors, such as Apelin-12, LAD, RVD, NT-proBNP, and NLRP3. These results demonstrate the significance of cardiac remodeling, inflammation and neurohormonal activation in the development of NDAF. Notably, the model allows early risk stratification and aids in individual clinical decision-making. This approach may support timely intervention and improve in-hospital management of high-risk patients with STEMI. More extensive cohort studies using long-term follow-up would be recommended to further support and generalize these findings. In addition, independent external validation is required to confirm the robustness, transportability, and clinical applicability of the proposed model before routine implementation.

## Data Availability

The datasets presented in this study can be found in online repositories. The names of the repository/repositories and accession number(s) can be found in the article/Supplementary material.
